# Mapping quantitative trait loci (QTL) in sheep. I. A new male framework linkage map and QTL for growth rate and body weight

**DOI:** 10.1186/1297-9686-41-34

**Published:** 2009-04-24

**Authors:** Herman W Raadsma, Peter C Thomson, Kyall R Zenger, Colin Cavanagh, Mary K Lam, Elisabeth Jonas, Marilyn Jones, Gina Attard, David Palmer, Frank W Nicholas

**Affiliations:** 1 ReproGen – Advanced Technologies in Animal Genetics and Reproduction, Faculty of Veterinary Science, University of Sydney, 425 Werombi Road, Camden NSW 2570, Australia; 2 Commonwealth Scientific and Industrial Research Organisation Plant Industry, Black Mountain, ACT 2601, Australia

## Abstract

A male sheep linkage map comprising 191 microsatellites was generated from a single family of 510 Awassi-Merino backcross progeny. Except for ovine chromosomes 1, 2, 10 and 17, all other chromosomes yielded a LOD score difference greater than 3.0 between the best and second-best map order. The map is on average 11% longer than the Sheep Linkage Map v4.7 male-specific map. This map was employed in quantitative trait loci (QTL) analyses on body-weight and growth-rate traits between birth and 98 weeks of age. A custom maximum likelihood program was developed to map QTL in half-sib families for non-inbred strains (QTL-MLE) and is freely available on request. The new analysis package offers the advantage of enabling QTL × fixed effect interactions to be included in the model. Fifty-four putative QTL were identified on nine chromosomes. Significant QTL with sex-specific effects (*i.e. *QTL × sex interaction) in the range of 0.4 to 0.7 SD were found on ovine chromosomes 1, 3, 6, 11, 21, 23, 24 and 26.

## Background

Over the past few decades, a number of quantitative trait loci (QTL) analyses have been conducted on many livestock breeds. These studies have provided very useful genetic information and enriched our knowledge on the underlying biology and genetic architecture of complex traits. A general review of QTL mapping can be found in Weller [1].

An important input to be considered in QTL studies is the availability of a robust framework map of the genome. The initial work by Crawford et al*. *[2] has resulted in the first extensive ovine genetic linkage map covering 2,070 cM of the sheep genome and comprising 246 polymorphic markers [3]. It has been followed by second [4] and third generation updates [5]. The latest update of the ovine linkage map has been recently published and is available on the Australian Sheep Gene Mapping website [6]. Several QTL studies have established independent linkage maps to position QTL, e.g*. *Beh et al*. *[7], Crawford et al*. *[8], Beraldi et al*. *[9], Murphey et al*. *[10] and Gutierrez-Gil et al*. *[11], using independent populations of Merino, Coopworth, Soay, Suffolk, and Churra sheep, respectively.

In sheep, growth rate and body mass represent economically important traits, which are under moderate genetic control and respond to directional selection [12]. Despite extensive background information, relatively few QTL studies have been reported for growth in sheep and furthermore they have been mostly restricted to partial genome scans, limiting the discovery of and reports on new QTL. QTL studies contribute to the understanding of the genetic basis of a biologically complex trait such as growth because they can identify positional candidate genes. Walling et al*. *[13] have reported QTL affecting muscle depth and live weight at eight weeks of age in Texel sheep from partial genome scans in candidate gene regions on *Ovis aries *chromosome 2 (OAR2) and OAR18. Using candidate regions on OAR1, 2, 3, 5, 5, 6, 11, 18 and 20 in Suffolk and Texel commercial sheep populations, Wallinget al*. *[13,14] have revealed suggestive QTL for body weight. Based on previous studies in sheep and other livestock species, McRae et al*. *[15] have analysed results of partial scans on selected autosomes (OAR1, 2, 3, 18 and 20) and identified QTL for body weight at eight and 20 weeks of age on OAR1. A whole genome linkage study, conducted in an Indonesian Thin Tail × Merino sheep population, has revealed QTL for birth weight on OAR5 and for body weight at yearling on OAR18 [16].

Combining results from QTL analyses in different livestock species and functional and positional candidate gene studies have shown that the myostatin gene on OAR2, the insulin-like growth factor-1 gene on OAR3, the callipyge gene and the Carwell rib eye muscling locus on OAR18 and the MHC locus on OAR20 are linked to growth or muscularity QTL in sheep and/or cattle [13,17-29]. However, incomplete genome scans and positional candidate gene studies give an incomplete picture of the whole genome and of the location of growth and body weight QTL.

In this paper, we report the development of a framework map for male sheep, derived from a paternal half-sib design within an Awassi × Merino resource population. We use this map to search for putative QTL for growth rate and body weight in this resource population. In subsequent papers, we will report other putative QTL for economically important production traits such as milk yield and milk persistency, fleece/wool production, carcass characteristics, reproduction, behaviour, feed intake, and type traits. The range of phenotypes collected during this study is listed in the additional file 1.

## Methods

### Resource population

As described by Raadsma et al*. *[30], a resource population from crosses between Awassi and Merino sheep was established to exploit the extreme differences between these two types of sheep in a range of production characteristics. Awassi sheep is a large-frame fat-tailed breed, which has its origins in the Middle East as a multi-purpose breed for milk, carpet wool and meat production and where it is dominant. From this source, the modern milking Awassi sheep was developed in Israel [31], which is the breed used in the present resource. Merino sheep is known for high-quality apparel wool but poor maternal characteristics [32]. The Australian Merino breed, which is dominant in Australia, was derived from Spanish and Saxon Merinos crossed with meat breeds imported from Capetown and Bengal [33]. Both super-fine and medium-wool Merinos were used in the present resource: they have a much smaller frame size than the milking Awassi breed and a very different fat distribution.

**Figure 1:**
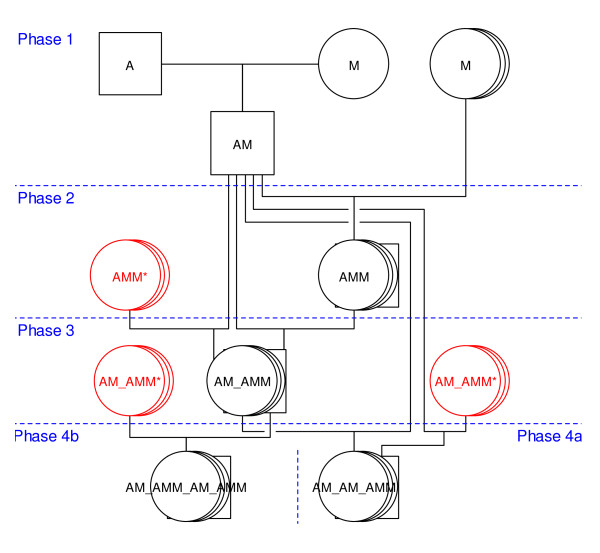
**Mating structure for a single sire family in the Awassi × Merino resource population**. A = Awassi, M = Merino; in Phases 3 and 4, ewes are brought in from other sire families, shown as the AMM* and AM_AMM*; the other three sire families have similar mating structures, again with cross-family matings in Phases 3 and 4.

This resource population was developed in three phases, coinciding with different stages of research. A diagrammatic representation of the mating structure is shown in Figure 1 for one of the sire families and the other families have similar mating structures. In Phase 1, four sires from an imported strain of improved dairy Awassi [31], were crossed with 30 super-fine and medium-wool Merino ewes. Four resulting F_1 _sires (AM) were backcrossed to 1650 fine and medium-wool Merino ewes, resulting in approximately 1000 generation-2 (G_2_) backcrosses (AMM). In Phase 2, 280 AMM G_2 _ewes were mated to the four AM F_1 _sires so that matings were both within family (F_1 _sire mated with his daughters) and across families (F_1 _sire mated with daughters of other F_1 _sires) to produce approximately 900 G_3 _animals (AM_AMM). In Phase 3, 280 of the available G_3 _ewes were mated to three of the AM F_1 _sires (both within and across sire families) to produce G_4a _animals (AM_AM_AMM). In addition, four G_3 _males (each replacing one of the F_1 _sires) were mated to G_3 _ewes, resulting in 490 G_4b _animals (AM_AMM_AM_AMM). A total of 2,700 progeny were produced over 10 years, representing four generations. A broad range of phenotypes was collected from the progeny, as well as a DNA and tissue (blood, milk, fat, muscle, wool) repository for each available animal. In the initial QTL study reported here, only phenotypic and genotypic information from the G_2 _backcross progeny of the first F_1 _sire were analysed in detail, as this was the only family where a genome-wide scan was performed. The additional families will be used for confirmation of QTL effects and, when combined with high-density marker analysis, for fine mapping of confirmed QTL.

Progeny were reared in typical Australian paddock conditions for a NSW Southern Tablelands environment. Supplementary feeding occurred at times when feed availability from pasture was limited and corresponded to periods of negative growth (approximately 12 months of age). From 83 to 98 weeks (at which time the growth study was terminated), only the males were maintained on pasture as a single cohort till separate feed intake and carcass studies were undertaken. Ewes were relocated to a separate farm for lambing and milk recording.

### Genotyping

DNA was extracted from blood using a modification of the protocol described by Montgomery and Sise [34]. Purity of all extracted DNA was assessed by calculating the 260/280 nm ratios determined with an Eppendorf BioPhotometer. All DNA samples were dispensed to 96-well plates using a robotic workstation (Beckman Biomek 2000 with integrated MJ research DNA Engine PCR cycler).

Two hundred previously published polymorphic microsatellite markers covering all 26 autosomes were used in the construction of the map. They comprised 112 cattle (*Bos taurus*) markers, 73 sheep (*Ovis aries*) markers, and 15 other bovidae markers sourced from Prof. Yoshikazu Sugimoto (pers. comm.). All markers were screened for phase-known heterozygosity for the sire genotype. Markers were chosen on their Polymorphic Information Content [35] (PIC; > 0.6 if possible), and ease of scoring. Five hundred and ten animals were genotyped, comprising the Awassi grandsire, the Merino grand dam, and 510 AMM backcross G_2 _progeny (246 ewes and 264 wethers).

PCR was performed in 10 μL reactions containing 50 ng DNA, 1 × PCR buffer, 1 × 2.5 mM MgCl_2_, 200 μM of each dNTP, 0.8 pmol of each forward primer (with M13-29 tail) and reverse primer, 0.2 pmol of M13-29 primer labelled with either IRD 700 or IRD800 dye, and 0.5 units of *Taq *polymerase. PCR amplifications were carried out using one of the following three MJ Research (Watertown, Massachusetts, USA) 96 well PCR machines, namely, PTC-100, PTC-200, and PTC-200 Gradient Cycler.

The touchdown program (*Licor-50*) was used for the majority of the PCR, and a second program (*Cav-low*) was used for markers with a lower annealing temperature if amplification was unsuccessful using the *Licor-50 *program. The *Licor-50 *thermocycler touchdown cycles were as follows: initial denaturation for 5 min at 95°C, 5 cycles of 95°C for 45 s, 68°C for 1.5 min (-2°C per cycle), 72°C for 1 min, followed by 4 cycles of 95°C for 45 s, 58°C for 1 min (-2°C per cycle), 72°C for 1 min, followed by 25 cycles of 95°C for 45 s, 50°C for 1 min, 72°C for 1 min and a final 5 min extension at 72°C. The *Cav-low *cycles were as follows: initial denaturation for 5 min at 95°C, 5 cycles of 95°C for 30 s, 55°C for 1.5 min, 72°C for 45 s, followed by 5 cycles of 95°C for 30 s, 50°C for 30 s, 72°C for 45 s and a final 5 min extensions at 72°C.

Microsatellite PCR products were separated by polyacrylamide electrophoresis (PAGE) and detected using a Licor 4200 semi-automated sequencer.

### Scoring of genotypes

The following description applies to the genotype scoring of the AMM backcross only as mentioned previously. All genotypes were scored by at least two independent scorers. To facilitate linkage analysis, only the F_1 _allele source was scored (Awassi or Merino origin), rather than the actual allele size. The Awassi allele was scored as '1', while the Merino allele was scored as '2', giving a genotype for the F_1 _sires. Only the identities of the alleles that were in the F_1 _sire were scored in the G_2 _AMM backcrosses, their genotypes identified as '1', '2' or '12'. A score of 1 can be homozygous '11' or 1*x*, where *x *is not equal to 2. Similarly a score of 2 can be homozygous '22' or 2*x*, where *x *is not equal to 1. Since information of the maternal allele was not available, heterozygous '12' in the backcross progeny was only semi-informative, as one cannot determine which allele originated from the F_1 _sire or from the Merino dam. The QTL mapping methodology used here exploited the semi-informative marker information (additional file 2).

### Sheep map

Using the genotype information from our Awassi-Merino resource population, we generated an independent sheep linkage framework map comprising the 200 microsatellites genotyped in this resource. Carthagene version 4.0 [36,37] and Multipoint [38] were used for the construction and validation of the map. These two programs use a multipoint maximum likelihood estimation method. Carthagene was used for the initial map construction, and Multipoint was used to test and validate marker orders. Only markers showing consistent results from both programs were included in the final framework map.

We used information from the Sheep Linkage Map v4.7 [6] to group markers according to their chromosomal location as a prior to the construction of the framework map. Marker ordering and validation were performed for each linkage (chromosome) group separately. A minimum LOD score of 3.0 and a maximum recombination fraction of 0.4 were used as thresholds for linkage and sub-linkage grouping within the same chromosome. The Kosambi map function [39] was used to convert recombination fractions to distances. A framework map was considered satisfactory for the marker positions within a linkage group if the LOD score difference between the best and next-best map order was greater than or equal to 3.0.

### Analysis of growth data

Non-fasted body-weight measurements were taken at weeks 2, 15, 25, 32, 37, 43, 48, 50, 56, 60, 67, 74, 79, and 83 for 510 G_2 _AMM backcrosses (246 ewes and 264 wethers). Birth weight was recorded for some animals, and body weights at weeks 90 and 98 were recorded for males only. The analysis of these data indicated distinct changes in growth rate at weeks 43, 56, and 86, presumably as a result of seasonal influences. Thus, growth rates were divided into four growth phases: week 0 to week 43, week 43 to week 56, week 56 to week 83, and week 83 to week 98. To accommodate these distinct changes, a piecewise-linear mixed model was used to model growth of each animal. Linear mixed models were fitted with separate slopes in each phase, but constrained to connect at each breakpoint (spline knot). While, arguably, a non-linear growth model may have been more applicable, the major purpose of the modelling was to capture the main features of the growth data. A full description of the piecewise-linear mixed model can be found in the additional file 2.

### QTL mapping procedure

A maximum likelihood procedure, named QTL-MLE, suitable for the backcross design of the present resource (in which only the paternal allele was identified in G_2 _animals) was developed and programmed using R [40] by one of us (PCT). The software allows easy modification for the identification of QTL for most types of traits, including binary (e.g*. *disease presence-absence), ordinal (e.g*. *5-point disease severity scale), or survival-time traits. Details of the algorithm are provided below, in terms of the models used to analyze body weight and growth data.

#### QTL-MLE algorithm

For a normally distributed trait, a linear model may be appropriate, *i.e*. *y*_*i *_= β'x_*i *_+ γ*q*_*i *_+ ε_*i*_, where *y*_*i *_= observed trait value of animal *i*, *i *= 1, ... *n*; x_*i *_= set of covariates and fixed effects for animal *i*; β = corresponding set of regression parameters; γ = sire family allelic QTL effect (*Q *relative to *q*); *q*_*i *_= unobserved QTL allele of animal *i*, = 1 if *Q*, 0 if *q*; and ε_*i *_= random error, assumed *N*(0,σ^2^). Note the Merino dam effects will be absorbed into this last term. The genotype of the F_1 _sire is assumed to be *Qq*, with *Q *originating from the Awassi line and *q *from the Merino line.

Since there are only two types of QTL alleles in backcross animals, the phenotype distribution is a mixture of two distributions. We calculate the QTL transmission probability (π_*i*_) as the probability of the sire transmitting QTL allele *Q *= π_*i *_= *p*(*q*_*i *_= 1 | m_*i*_), while the probability of transmitting the other allele *q *is 1 - π_*i *_= *p*(*q*_*i *_= 0 | m_*i*_), where m_*i *_is the "flanking" marker genotype information. Probabilities depend on the distance from the putative QTL to the marker(s) calculated via Haldane's mapping function. If the immediate flanking markers are "informative" (genotyped as '1' or '2'), they provide all possible information. Wherever a "semi-informative" marker ('12') is encountered adjacent to a putative QTL, the minimal set of markers that contains all the information for that QTL comprises the smallest set of contiguous markers flanked by "informative" markers.

At regular distances (typically 1 cM) along the length of the chromosome, the log-likelihood is constructed assuming a QTL at that position (*d*), *i.e.*



where *f*(·) is the probability density function (PDF) for a normal distribution (assuming that is the appropriate model for the data type). The log-likelihood is maximized using the E-M algorithm[41], which allows standard linear model software to be used, in an iterative manner. This requires computation at each iteration of the posterior probabilities (τ_*i*_) that the sire transmits allele *Q*, conditional on its phenotype,



At the peak log-likelihood position (*i.e. *estimated QTL location), these τ_*i *_values can be used to classify backcross animals with high probability of having received the *Q *(or *q*) allele. Also at the peak, a 1-LOD support interval for estimated QTL position was determined by determining the range of map positions that are within one LOD of the peak.

Implementation of the program in R has the advantage that the QTL mapping procedure can be extended within other modelling and graphical capabilities of this package. For normally distributed traits, the linear model function lm() is used, and this easily allows model extension to include interactions between the QTL and other fixed effects, such as sex-specific QTL effects: most other QTL analysis programs do not allow such extensions. Another advantage of the R system is the relative ease to model traits of different types. This is achieved by changing only a few lines of code, primarily (1) replacing the lm() call by another function call, and (2) replacing the normal PDF in the τ_*i *_calculation (dnorm()) by the appropriate PDF (or discrete probability function) for the required distribution.

Using QTL-MLE, separate genome scans were conducted for single QTL on the bodyweights at the start and end of the four growth phases. For these traits, the model-based predictions from the piecewise-linear mixed model output were analysed rather than the raw data. The stages analysed were at weeks 2, 43, 56, 83, and 98. Note that week-2 bodyweights were selected in preference to week-0 (start of Phase I) due to the relatively few birth weights available. The model fitted to these values was as follows:



where

Weight_*i *_= model-based bodyweight at week *i *(2, 43, 56, 83, and 98);

Sex = 1 if ram/wether; 0 if ewe;

QTL = 1 if Awassi allele, *Q*; 0 if Merino allele, *q *(allele type is unobserved); and

ε = residual random error term.

Note that the unobserved QTL term is taken into account using the E-M algorithm of the interval mapping procedure. The interaction term was added to allow for sex-specific QTL effects.

Similarly, the average growth rates during each growth phase were analysed as separate traits. Again, model-based growth rates were used, as obtained from the piecewise-linear mixed model, and the model-based bodyweight at the start of each growth phase was used as a covariate. (As in the growth rate QTL model, the week-2 predicted bodyweights were used in preference to week-0 predicted ones). The model fitted for this QTL analysis took the following form:



where

GR_*i *_= model-based average growth rate in growth phase *i *and

Weight_*i *_= model-based bodyweight at start of growth phase *i*.

Since data for only wethers were available for the last growth phase (83–98 weeks), a term for sex was not included in either the week-98 body weight analysis, or the growth rate analysis. An additional series of analyses was performed without inclusion of the initial weight as a covariate.

Because of the large number of analyses, we adopted the false discovery rate (FDR) method of Benjamini and Hochberg [42] to adjust *P*-values for all traits to control for genome-wise error rates. Results were concluded to be significant when the adjusted *P*-values were less than 0.05. In all of these cases, LOD scores generated by QTL-MLE were larger than 2; QTL are described as suggestive where the *F*-value exceeds chromosome wide *P *< 0.05 threshold but not the 0.01 threshold. Based on a type I error of 0.01, the design had a power of 0.80 to detect QTL with 0.3 SD effect with 510 animals and an average marker spacing of 20 cM [43].

#### QTL mapping using QTL Express

For comparative purposes, all traits were analysed using the half-sib applet in QTL Express [44]. With the exception of the QTL × fixed effect interaction, the same fixed effects as in the MLE analysis were fitted. Chromosome-wide significance thresholds were assessed using permutation tests [45], and bootstrap procedures [46] were used to obtain confidence intervals, both implemented in QTL Express using 1,000 re-samplings.

Methods for mapping a single QTL can be biased by the presence of other QTL [47,48]. To address this situation, two-QTL models were also fitted for all traits using QTL Express [44]. To control for false-positive QTL due to multiple testing, the permutation thresholds obtained in the single-QTL analyses were used to test for the significance of the two-versus one-QTL for a particular trait. Corresponding *F*-values for the two-versus zero-QTL test are included for comparison and additional support, although the same significance thresholds would not be applicable (given it would be a two numerator df test rather than a one df test).

## Results

### Sheep framework map

From the 200 markers used, 194 markers showed significant linkage with at least one other marker at a LOD score of 3 or greater within their assigned linkage group (chromosome). The six markers that did not show significant linkage with other markers on their assigned chromosome were DIK4933 and OARFCB129 on OAR3, TGLA116 on OAR4, MCM185 on OAR7, BM6108 on OAR10 and RM024 on OAR24. All these markers were excluded from the framework map. A further three markers were excluded because their inclusion did not improve the overall LOD score of the framework map, even though they had a LOD of 3 or greater with one other marker within their linkage group. These three markers were KAP8 on OAR1, TGLA67 and OARFCB5 on OAR3. The final map contains 191 markers.

For the framework map, both Carthagene and Multipoint produced the same linkage and map order results. The additional file 3 presents the LOD score differences between the best and second-best map order for each chromosome generated by Carthagene. Except for OAR1, 2, 10 and 17, all other chromosomes yield a LOD score difference greater than 3.0 between the best and second-best map order. Thus the framework map can be considered fixed for the majority of the chromosomes. A detailed higher resolution order and length can be found in additional file 4.

In our framework map, we have also included four bovine microsatellite markers (DIK4572, DIK4527, DIK4612, and DIK2269) that are presently not included on the Sheep Linkage v4.7 Best Position Map. DIK4572 has been mapped to BTA2 [49] and in the present study is placed on OAR2 with a two-point LOD score of 4.8 with its closest marker INRA135. DIK 4527, DIK4612 and DIK2269 all map on BTA20 [49], and in the present study are placed on OAR16 with respective two-point LOD scores of 28.2, 14.7 and 11.8 with their closest neighbouring markers. These bovine and ovine positions are consistent with the cattle-sheep comparative map as shown on the Sheep Linkage Map web site .

Apart from a slight difference in marker position, the marker order of the ReproGen Framework Map is the same as the Sheep Linkage Map Best Position Map v4.7. Sixteen chromosomes had a length at least a 7 cM greater than that in Sheep Linkage Map v 4.7, indicating slightly more recombination in the ReproGen map population. Six chromosomes (OAR4, 6, 12, 13, 23, 26) showed a similar length (within 3 cM) in both maps.

### Overall growth performance

**Figure 2:**
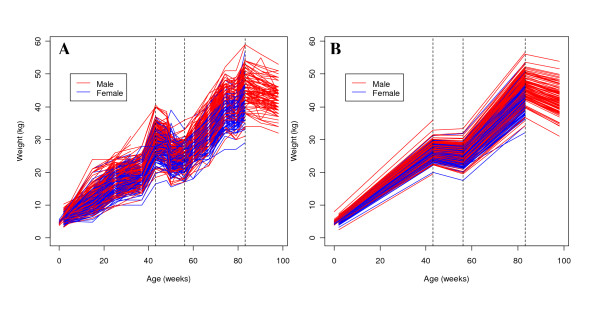
**Plot of body weight over time**. (A) Raw body weight data; (B) predicted values after piecewise-linear mixed modeling; the three dashed vertical lines separate the four growth phases at 43, 56, and 83 weeks.

Table 1 presents the number of observations, the mean and the standard deviation of body weight at each of the measurement weeks. The plot of the weights (Figure 2A) indicates distinct changes at weeks 43, 56, and 86, suggesting growth phases. The fitted piecewise-linear mixed models for individual sheep are shown in Figure 2B.

**Table 1:** Descriptive statistics of body weight (kg) at different ages

Trait^a^	N	Mean	St Dev
BW00	84	4.20	0.83
BW02	514	5.22	1.33
BW15	406	11.53	2.61
BW25	409	17.31	2.86
BW32	21	22.90	3.41
BW37	385	19.24	2.68
BW43	385	29.07	3.51
BW48	385	28.13	3.31
BW50	384	24.26	3.14
BW56	380	24.80	2.94
BW60	377	27.68	3.11
BW67	374	32.70	3.81
BW74	371	40.19	3.89
BW79	372	40.13	3.90
BW83	372	43.82	4.69
BW90	91	44.00	4.20
BW98	91	42.29	3.91

^a^Traits are shown as BW*xx *where *xx *is the age in weeks

All fixed effect terms in the piecewise-linear mixed model are significant (Table 2) indicating different growth profiles for both sexes, and support for the change in growth rate across the four phases. Table 2 also shows the estimated variance components, with their approximate standard errors. These represent individual animal variation in birth weights, and also in their individual growth rates, across the different phases.

**Table 2:** Summary of results of analysis with the piecewise-linear mixed model

Fixed effect	DF	F	P
Sex	1	10.23	0.0014
GR00-43	1	16115.39	< 0.0001
GR43-56	1	18.93	< 0.0001
GR56-83	1	391.35	< 0.0001
GR83-98	1	959.88	< 0.0001
Sex × GR43-56	1	31.79	< 0.0001
Sex × GR56-83	1	16.33	< 0.0001
Sex × GR83-98	1	8.51	0.0035

Random effect		Variance	Z*

Animal		0.683	5.91
Animal × GR00-43		1.33 × 10^-3^	9.20
Animal × GR43-56		9.08 × 10^-4^	2.20
Animal × GR56-83		3.51 × 10^-3^	5.09
Animal × GR83-98		2.08 × 10^-3^	0.66
Residual		6.156	35.97

The first half of the table shows the fixed effects, and the second half shows the random effects (variance components); GR*xx-yy *refers to the growth rate in the interval *xx-yy *weeks, expressed as a change from the growth rate in the previous interval; see additional file 2 for model details; the *F *statistics are incremental ones, *i.e. *testing the effect of that term, given the previous terms included in the model, *Z = estimated variance component/SE of its estimate; values greater than 2 can be considered 'significant'

### Putative QTL identified for growth rate and body weight

#### Single QTL Analysis

**Figure 3:**
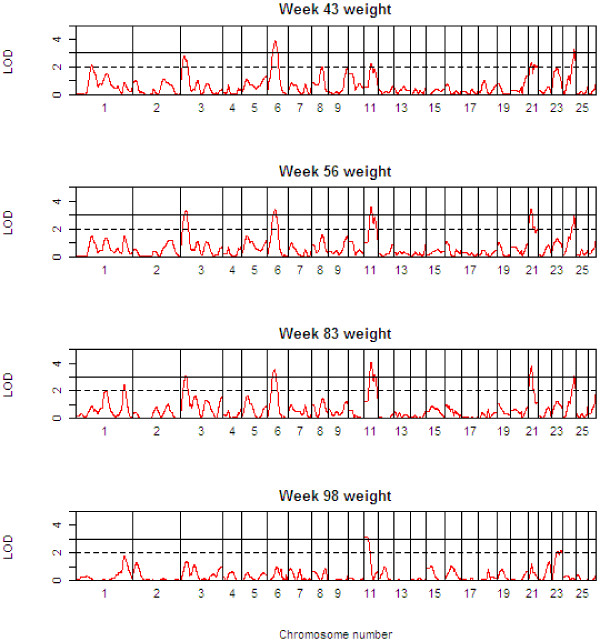
**QTL Map of the entire genome for body weight traits (BW*xx*)**.

**Figure 4:**
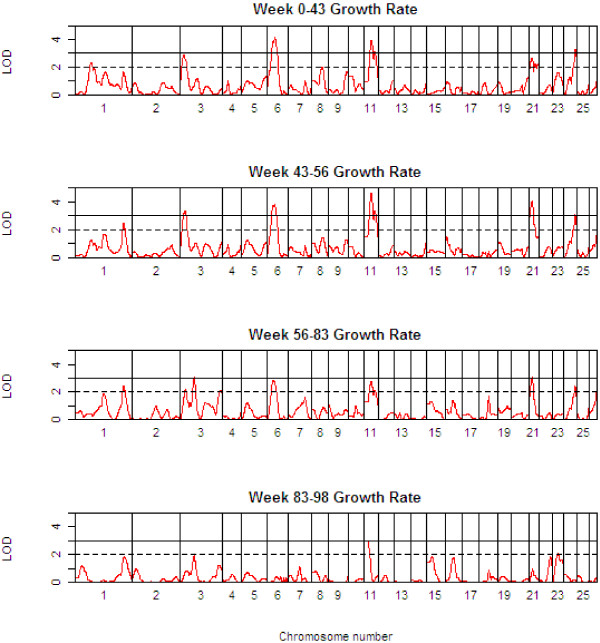
**QTL Map of the entire genome for growth rate traits (GR*xx*-*yy*)**.

**Figure 5:**
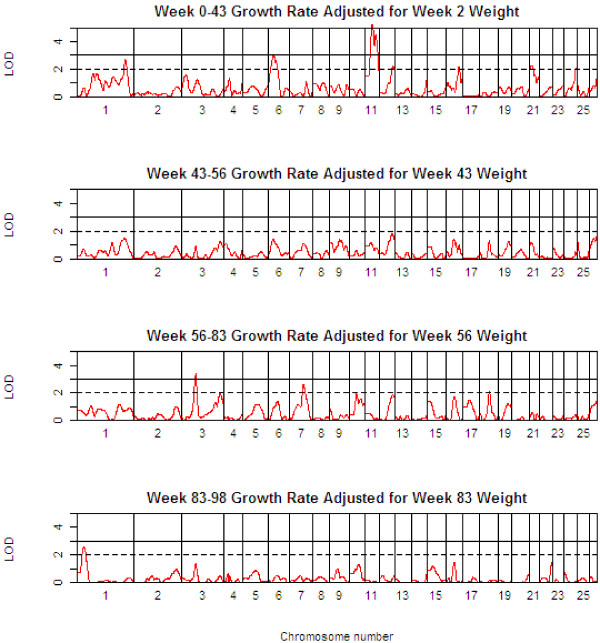
**QTL Map of the entire genome for growth rate traits adjusted for initial body weight (GR*xx*-*yy *adj *xx*)**.

Table 3 presents detailed results of the genome scan for QTL of body weight (BW) at the critical weeks separating the growth phases. Table 4 shows the corresponding information for growth rate (GR) during each of the four phases, whilst Table 5 shows the same information for growth rate traits, but after adjustment for body weight at the start of the growth phase. The 1-LOD support intervals generated by QTL-MLE are also reported. Figure 3 presents a QTL map showing the alignment of the QTL for all body weight traits along the genome, and Figures 4 and 5 show similar scans for growth rate QTL, unadjusted and adjusted for initial body weights. The additional file 5 contains all results using QTL-MLE and QTL Express showing the relative positions of the peaks along the genome for the different traits.

**Table 3:** Summary of QTL results for body weights at weeks 2, 43, 56, 83, and 98

Body Weight Trait	OAR	LOD with Ranked *P*-value#	QTL Position	Closest Marker	Std QTL Effect for Female	Std QTL Effect for Male	Sex × QTL Interaction *P*-value	1-LOD Support Interval
BW02	NS							

BW43	1	2.13*	117	BM4129	0.077	0.596	0.037	95.5 – 150.5
	3	2.84*	29	OARCP34	-0.145	0.722	0.001	12.0 – 55.0
	6	3.90***	63	OARHH55	0.187	0.749	0.025	43.9 – 82.9
	11	2.25*	55	BM17132	0.248	0.703	0.115	40.3 – 95.3
	21	2.31*	29	CSSM13	0.063	0.532	0.306	7.0 – 78.0
	24	3.26**	85	DIK2568	-0.150	0.584	0.002	73.1 – 98.1

BW56	3	3.32***	42	OARCP34	-0.140	0.665	0.0007	18.0 – 54.0
	6	3.45***	62	OARHH55	0.263	0.687	0.113	40.9 – 80.9
	11	3.56***	55	BM17132	0.338	0.824	0.077	42.3 – 68.3
	21	3.44***	27	CSSM13	0.047	0.667	0.010	8.0 – 36.0
	24	2.91**	85	DIK2568	-0.126	0.553	0.008	67.1 – 98.1

BW83	1	2.48*	357	BMS1789	-0.010	-0.629	0.011	348.5 – 375.5
	3	3.11***	42	OARCP34	-0.098	0.665	0.006	20.0 – 54.0
	6	3.53***	60	OARHH55	0.382	0.626	0.355	38.9 – 75.9
	11	4.03***	55	BM17132	0.348	0.898	0.047	43.3 – 67.3
	21	3.79***	29	CSSM13	0.083	0.677	0.008	12.0 – 35.0
	24	3.06***	85	DIK2568	-0.103	0.587	0.005	71.1 – 97.1
	26	2.11*	63	OARJMP23	0.042	0.480	0.038	50.0 – 62.5

BW98	11	3.10***	29	HEL10	NA	0.870	NA	29.3 – 46.3
	23	2.15*	71	URB0031	NA	0.843	NA	22.5 – 83.0

BW*xx *is the body weight at week *xx*; models fitted are of the form 'Sex + QTL + Sex.QTL', except for week 98 (wethers only) for which the model was 'QTL' only; *P*-values were obtained from likelihood ratio tests (LRT) with 2 df (testing QTL + Sex.QTL) or 1 df (QTL only, in Week 98); these *P*-values were adjusted for false discovery using the method outlined in Benjamini and Hochberg [42] with *P < 0.05, **P < 0.001, ***P < 0.005, ****P < 0.0001; standardized QTL effects are expressed as the estimated effect difference (Awassi – Merino) relative to the estimated residual standard deviation

**Table 4:** Summary of QTL results for growth rates across different phases of growth

Growth Rate Trait	OAR	LOD with Ranked *P*-value	QTL Position	Closest Marker	Std QTL Effect for Female	Std QTL Effect for Male	Sex × QTL Interaction *P*-value	1-LOD Support Interval
GR00-43	1	2.33*	118	BM4129	0.048	0.624	0.019	99.5 – 153.5
	3	2.87**	28	OARCP34	-0.165	0.737	0.001	11.0 – 52.0
	6	4.16***	62	OARHH55	0.242	0.767	0.029	38.9 – 78.9
	8	2.02*	77	KD101	0.199	-0.584	0.034	56.4 – 94.4
	11	3.96***	55	BM17132	0.305	0.905	0.028	44.3 – 67.3
	21	2.62*	26	CSSM13	0.109	0.584	0.160	5.0 – 78.0
	24	3.30***	86	DIK2568	-0.158	0.599	0.001	80.1 – 100.1

GR43-56	1	2.47*	357	BMS1789	-0.038	-0.622	0.015	348.5 – 376.5
	3	3.33***	39	OARCP34	-0.092	0.643	0.001	17.0 – 51.0
	6	3.86***	60	OARHH55	0.350	0.679	0.228	36.9 – 75.9
	11	4.60****	54	BM17132	0.346	0.970	0.022	43.3 – 64.3
	21	4.04***	27	CSSM13	0.091	0.716	0.007	10.0 – 35.0
	24	3.14***	85	DIK2568	-0.096	0.587	0.003	76.1 – 97.1
	26	2.00*	63	OARJMP23	0.056	0.461	0.054	49.0 – 62.5

GR56-83	1	2.44*	357	BMS1789	-0.039	-0.620	0.016	347.5 – 379.5
	3	3.05*	105	DIK4796	0.015	0.575	0.033	94.0 – 116.0
	6	2.84*	50	BM1329	0.493	0.428	0.794	33.9 – 75.9
	11	2.77*	53	BM17132	0.298	0.732	0.126	37.3 – 70.3
	21	3.08*	29	CSSM13	0.108	0.597	0.030	12.0 – 35.0
	24	2.47*	85	DIK2568	-0.053	0.538	0.010	69.1 – 98.1
	26	2.37*	63	OARJMP23	0.147	0.483	0.110	49.0 – 62.5

GR83-98	11	2.95**	29	HEL10	NA	0.860	NA	29.3 – 43.3
	23	2.06*	40	MCMA1	NA	0.901	NA	20.5 – 83.0

Growth rates are not adjusted for the body weight at the start of the phase; GR*xx-yy *refers to the growth rate in the interval *xx-yy*; models fitted are of the form 'Sex + QTL + Sex.QTL', except for week 98 (wethers only) for which the model was 'QTL' only. *P*-values were obtained from likelihood ratio tests (LRT) with 2 df (testing QTL + Sex.QTL) or 1 df (QTL only, in week 98); these *P*-values were adjusted for false discovery using the method outlined in Benjamini and Hochberg [42] with *P < 0.05, **P < 0.001, ***P < 0.005, ****P < 0.0001; standardized QTL effects are expressed as the estimated effect difference (Awassi – Merino) relative to the estimated residual standard deviation

**Table 5:** Summary of QTL results for growth rates across different phases of growth

Growth Rate Trait	OAR	LOD with Ranked *P*-value	QTL Position	Closest Marker	Std QTL Effect for Female (A-M)^†^	Std QTL Effect for Male (A-M)^†^	Sex × QTL Interaction *P*-value	1-LOD Support Interval
GR00-43 adj for BW02	1	2.75*	357	BMS1789	-0.085	-0.707	0.022	346.5 – 373.5
	6	3.06**	43	BM1329	0.387	0.590	0.425	21.9 – 72.9
	11	5.23***	56	BM17132	0.328	0.959	0.017	45.3 – 67.3
	12	2.20*	104	HUJ625	-0.232	0.561	0.003	84-3 – 118.0
	16	2.19*	98	DIK4612	-0.148	-0.454	0.166	85.0 – 110.0
	21	2.27*	19	CSSM013	0.203	0.542	0.162	0.0 – 53.0
	24	2.05*	91	DIK2568	-0.158	0.494	0.009	80.1 – 104.7

GR43-56 adj for BW43	NS							

GR56-83 adj for BW56	3	3.41***	105	DIK4796	0.151	0.584	0.040	96.0 – 113.0
	7	2.62*	100	TGLA444	0.524	-0.124	0.017	90.0 – 122.2
	18	2.14*	72	BM7243	-0.100	0.474	0.009	61.1 – 85.1

GR83-98 adj for BW83	1	2.59*	48	BMS835	NA	0.871	NA	32.5 – 67.7

Growth rates are adjusted for the body weight at the start of the phase. GR*xx-yy *refers to the growth rate in the interval *xx-yy *and BW*xx *is the body weight at week *xx; *Models fitted are of the form 'BW*xx *+ Sex + QTL + Sex.QTL', except for week 98 (wethers only) for which the model was 'BW*xx *+ QTL' only; *P*-values were obtained from likelihood ratio tests (LRT) with 2 df (testing QTL + Sex.QTL) or 1 df (QTL only, in Week 98); these *P*-values were adjusted for false discovery using the method outlined in Benjamini and Hochberg [42] with *P < 0.05, **P < 0.001, ***P < 0.005, ****P < 0.0001; standardized QTL effects are expressed as the estimated effect difference (Awassi – Merino) relative to the estimated residual standard deviation

With the exception of BW02, QTL for body weight traits have been identified across the sheep genome (OAR1, 3, 6, 11, 21, 23, 24, and 26). Importantly, examination of the 1-LOD support intervals suggests that the same QTL are involved in various body weight traits (OAR3 for BW43, BW56, and BW83, OAR6 for BW43, BW56, and BW83, OAR11 for BW43, BW56, and BW83, OAR21 for BW43, BW56, and BW83 and OAR24 for BW43, and BW83). In addition, the QTL effects for males were almost always greater in absolute value than for females, and for males in particular, the effect of the Awassi allele led to an increase in body weight relative to the Merino allele.

Multiple QTL were also detected for the growth rate traits, and in general, these correspond to the QTL identified for the critical body weight traits, in terms of map position and also effect. All the body weight QTL also mapped to growth rate QTL, but in addition a suggestive QTL was found on OAR8 for GR00-43. While the growth rate QTL are in general the same as the body weight QTL, the analysis of growth rate QTL adjusting for the body weight at the start of the growth phase shows quite different results. Note that for the first growth phase, the body weight covariate adjusted for was BW02, since there were relatively few animals with birth weights data. After adjusting for initial body weight, QTL were identified for the first growth phase, GR00-43, corresponding to many of the regions previously identified for body weight and unadjusted growth rate traits, and an additional suggestive QTL was mapped on OAR16. However, no QTL were detected for GR43-56 after adjusting for BW43 (this period corresponding to a period of weight loss). Three QTL (on OAR3, 7 and 18) were detected for GR56-83, and only one QTL (on OAR1) for GR83-98.

Note that OAR1 is involved in body weight and growth rate QTL on three chromosomal locations, namely 32–68 cM (GR83-98 adj for BW83, positive effect of Awassi allele), 95–154 cM (BW43, GR00-43, both positive effects), 346–380 cM (BW83, GR43-56, GR56-83, GR00-43 adj for BW02, all negative effects).

Mapping results obtained by QTL Express were consistent with those obtained by QTL-MLE, particularly for those with greater effects (additional file 5). QTL Express also identified additional QTL on OAR6, 16 (GR02 in week 2) and OAR3 and 26 (GR4 in week 42) (but as noted earlier, it was not possible to fit sex-specific QTL effects in QTL Express).

### Two-QTL analysis

Significant results for the two-QTL model are presented in Table 6. Overall, the two-QTL procedure detected far fewer QTL compared with the single-QTL methods, as QTL were detected for only three traits. For adjusted GR56-83, two QTL were detected in coupling phase on OAR3, one at 104 cM and the other at 284 cM, both with effects of ~0.35 SD (Table 6), the 3D surface plot of the variance ratio test statistic is shown in the additional file 6. In the single-QTL analyses, the second peak at 284 is clearly visible on the interval map. For the adjusted GR00-43, two QTL were mapped in very close proximity on OAR4 (108 cM and 112 cM) in repulsion phase. However, given that both these positions are flanked by the same markers (OARCP26 and OARHH35), and that both QTL have extremely large estimated effects (~ ± 2.8 SD), it is unlikely that these are real QTL. For adjusted GR83-98, a similar situation occurred on OAR4 (24 cM and 28 cM), and on OAR22 for the same trait (68 cM: -1.75 SD, and 88 cM: +1.90 SD) with the QTL being mapped to separate marker bracket intervals.

**Table 6:** Summary of two-QTL analysis results

Trait	OAR	*F*(2 vs 0)^1^	*F*(2 vs 1)^2^	Pos	Std effect^3^	Pos	Std effect
GR00-43 Adj BW2	4	6.16	8.49	108	-2.77	112	2.88

GR56-83 Adj BW56	3	9.98	8.16	104	0.34	284	0.37

GR83-98 Adj BW83	4	5.28	8.54	24	-3.58	28	3.52
	22	7.46	8.1	68	-1.75	88	1.90

^1^*F*(2 vs 0) is *F*-statistic for testing two QTL vs no QTL on chromosome

^2^*F*(2 vs 1) is *F*-statistic for testing two QTL vs one QTL on chromosome

^3^Std effect (standardized QTL effect) = QTL Effect/Residual Std Dev

## Discussion

This paper reports the construction of a male distance framework map for sheep and its application in the identification of QTL for body weight and growth. There are several advantages in developing a separate framework map. First, it provides an independent verification to the Sheep Linkage Map v4.7, since it originates from a single sheep resource. It would be possible to integrate the data in this map with the data of other Sheep Linkage Maps to create an integrated framework map for sheep. The ReproGen framework map and the Sheep Linkage Map v4.7 agree well, with no changes in marker order. With the exception of OAR4, 6, 12, 13, 14, 18, 23 and 26, the ReproGen map is 11% larger than the male-averaged Sheep Linkage Map v4.7. Perhaps one explanation for this is that the current map was constructed using a single sire family with a large number of progeny, compared with the Sheep Linkage Map v4.7, which is derived from a large number of smaller families. The second advantage in constructing an experiment specific framework map is that the QTL can be unambiguously mapped to a fixed location since the markers are in a fixed order. The use of a framework map not only allows integration of markers in a consensus map, but also alignment of QTL in integrated maps for future meta-analyses such as those undertaken in dairy cattle by Khatkar et al. [50].

The pattern of growth in this flock is consistent for sheep maintained on semi-improved pasture in a temperate Australian tablelands climate. Such grazing systems are characterized by low pasture availability in the colder winter months, and abundant pasture growth in spring with a residual pasture carry over in summer and autumn. The availability of pasture is reflected in the growth curve over a period of 52 weeks, with a rapid growth following birth in spring till the end of autumn, a period of no growth or decline coinciding with winter, and a subsequent cycle between 52 and 98 weeks.

Consistent with findings in previous studies using European sheep, we have identified a number of significant QTL for body weight and growth rates on different chromosomes. In order to minimize the large number of possible QTL detected for single-point estimates of body weight, all data were combined through a growth curve over a period where changes in body weight were similar for all sheep as shown in the stick-point graph (Figure 2B). The points of change in growth (break points) were identified by single-point body weights, which were used in the QTL analyses as reference body weights. Since growth between each break point was strongly influenced by starting body weights at each time, true growth rate was analysed by adjusting for starting body weights. The final outcome of summarizing all body weights in relatively few growth and body weight indicators was that 17 body weight time points were collapsed to 12 core traits instead of the 49 possible correlated traits. It allows for a greater consistency in QTL reporting for traits related to growth.

Despite the economic importance of body weight and growth rates in sheep and the extensive studies reporting genetic variation in this trait [12,51,52], relatively few QTL studies have been reported to date. In this study, we report 54 QTL involved in body weight and growth rate. The majority of the QTL (49 out of 54) are co-located across nine chromosomal regions, suggesting QTL with a general effect on growth and body weight (OAR1, 3, 6, 11, 21, 23, 24 and 26). Only five QTL (OAR7, 8, 12, 16 and 18) were observed for a single growth trait, and notably four of these were for growth rate adjusted for starting body weight. Our study identified new QTL for body weight/growth (OAR6, 7, 8, 11, 12, 16, 21, 24 and 26) since our study covers a full autosomal genome scan as distinct from the previous partial genome scans, to the best of our knowledge. Our study confirmed QTL for growth and body weight previously reported by Walling et al*. *[13,14], and McRae [15] and slaughter live weight [53] on OAR1, 3, and 18. In our study, no QTL for growth was observed on OAR2, and OAR5 where Walling et al*. *[29], Karamichou et al*. *[53] and Margawati et al*. *[16] reported QTL.

The effects of the QTL detected in this study are relatively large (in the order of 0.4 to 0.7 phenotypic standard deviations) and originated predominantly from the larger-framed Awassi grandsire, as expected with the favourable allele for growth and body size. In five out of 54 cases, the reverse was observed with the favourable allele originating from the Merino breed, suggesting the presence of cryptic QTL. These estimated QTL effects are consistent with the significant genetic variation in growth that has been observed within this breed (e.g*. *Merino body weight heritabilities are in the range of 0.39–0.76, based on a recent comprehensive study by Huisman et al*. *[52]. The current study has the power to detect QTL effects of 0.3 SD or larger. However, surprisingly for most traits, significant sex by QTL interactions were observed, with most QTL expressed in males but not females (Tables 3, 4, 5). No immediate or obvious explanation can be given for this, since the ewes and wethers were managed together for most of the period. A notable feature of this study was that the QTL locations have relatively small 1-LOD intervals, most likely due to the relatively large family size. This will facilitate positional candidate gene analyses and make a significant contribution to possible future meta-analyses. Some of the QTL reported in this paper are consistent with possible candidate genes that have been suggested for growth and body weight. The additional file 7 shows a comparison between the QTL region detected here with linkage and association studies of other groups as well as possible candidate genes possibly included in the regions.

The maximum likelihood approaches to QTL detection employed in this analysis were in general robust and their results were in good agreement with those of QTL Express [44], which is based on the least-squares methodology. Overall, 33 QTL were detected by both methods, with an additional 20 QTL identified by QTL-MLE alone, and another nine detected by QTL Express alone. However of the additional 29 QTL detected by only one of these methods, the majority (15/20 for QTL-MLE and 9/9 for QTL Express) had suggestive significance levels, as indicated by *P*-values for each method. However, it should be pointed out that the QTL-MLE could detect sex-specific QTL, which may have increased the power of the analysis. This is considered a notable advantage of our approach in that QTL × fixed effects can be fitted, which at the time of development was not possible in QTL Express. Obviously, this is a major consideration in our study since most QTL for growth and body weight appear to have differential effects in both sexes. An additional benefit of QTL-MLE is the flexibility of analysis offered, due to working in the R environment, with the ready potential for further enhancements in the future. However, QTL Express has the benefit of being able to fit two-QTL models. While three chromosomes containing two QTL were identified, two were unlikely given the size of the effect estimates, but strong support is indicated for two QTL for growth rate between weeks 56 and 83, adjusted for week-56 body weight on OAR3.

## Conclusion

In conclusion, we present significant evidence for an independent framework map, which is in very good agreement with the previously published Sheep Linkage Map v4.7 framework map for sheep. We also present evidence for a significant number of new QTL for body weight and growth rate in sheep, and confirm and support the presence of previously published QTL in breeds other than those studied here.

As indicated earlier, a large range of traits have been recorded from this sheep mapping study. Subsequent papers will describe QTL for these traits, using the current paper as a foundation to describe the overall approach. As SNP chips become more widely available for research in sheep genetics, this series of papers should provide a reference point to QTL studies in sheep using microsatellite markers.

## Competing interests

The authors declare that they have no competing interests.

## Authors' contributions

HR drafted the overall design, did the project management, and was involved in analysis of the data and writing the manuscript. PT developed the statistical methodology for the growth trait analysis and QTL methodology, implemented the QTL-MLE program, and contributed to manuscript preparation and the overall design. KZ and CC carried out the genetic marker analysis, genetic map construction, and ran the early stage analyses. ML ran the early stage QTL analyses, was responsible for the data assembly, and participated in the growth curve analyses. EJ helped run the QTL and data analyses, participated in the manuscript preparation and final response to referees. MJ and GA performed the genetic marker analyses, and did the DNA bank assembly. DP participated in the analysis, he further collected and provided the phenotypes and was responsible for the experimental animals. FN supervised the study, coordinated the design and initiation of the project and contributed to the manuscript preparation. All authors read and approved the final manuscript.

## Supplementary Material

Additional file 1 **Traits measured in the Awassi-Merino resource flock**. A list of all traits included the traits used for the analysis in the present paper, which were recorded in the sheep resource population used in the present study. Click here for file

Additional file 2 **Methodology QTL-MLE**. A detailed description of the QTL-MLE methodology which was developed by PCT and was used for the analysis shown here. Click here for file

Additional file 3 **LOD score difference between the best and second best map order**. This tables shows the differences of the LOD scored between the best and the second best map order, shown are the results on each autosome. Click here for file

Additional file 4 **Marker information for the Awassi × Merino map**. The figures shown here summarize the markers used in the study and compare the framework map described in the Awassi × Merino resource population with the published map. Further the identified linkage regions from the present study and QTL and candidate genes from various references are shown. All figures were designed using the MapChart software described by Voorrips R.E. in 2002 (MapChart: Software for the graphical presentation of linkage maps and QTLs. The Journal of Heredity 93 (1): 77–78). Click here for file

Additional file 5 **Summary of results using QTL-MLE and QTL Express. Values in table are estimated QTL positions (cM)**. This table shows the average QTL positions of all QTL presented here using both programs QTL-MLE and QTL Express. It summarizes and compares all results observed in the resource population for the growth and weight traits. Click here for file

Additional file 6 **3D surface plot of the variance ratio test statistic for a two-QTL model**. This figure shows the 3D surface plot for a two-QTL model for GR56-83 adj for BW56 on OAR3. The values of the plot are generated by QTL Express. Click here for file

Additional file 7 **Comparison of the QTL analysis results in the present studies with other results and possible candidate genes**. This table describes QTL identified in other studies which are located in comparable regions to the linkage regions identified using animals of the Awassi × Merino population. Further possible candidate genes are described. The list of references for these data is attached following the table. Click here for file
