# Supplementary material for: Mapping quantitative trait loci (QTL) in sheep. I. A new male framework linkage map and QTL for growth rate and body weight
Source: Genet Sel Evol. 2009 Apr 24;41(1):34. doi: 10.1186/1297-9686-41-34 (PMC2686678; doi:10.1186/1297-9686-41-34)
Supplement: Additional file 1 — Traits measured in the Awassi-Merino resource flock. A list of all traits included the traits used for the analysis in the present paper, which were recorded in the sheep resource population used in the present study. [file 1297-9686-41-34-S1.doc]

Additional file 1- Traits measured in the Awassi-Merino resource flock

| Trait |  |  | Trait |  |
| --- | --- | --- | --- | --- |
| Tail | Length |  | Fleece | Weight at 24 weeks |
|  | Weight |  |  | Weight at 50 weeks |
|  | Color score % |  |  | Weight at 75 weeks |
| Horn and Ear | Left and right horn length |  |  | Weight at 101 weeks |
|  | Left and right ear length |  |  | Merino fleece type at 24 wks |
| Pigment | Skin pigment |  |  | Merino fleece type at 50 wks |
|  | Nose pigment |  |  | Fleece rot at 24 wks |
|  | Fiber pigment |  |  | Fleece rot at 50 wks |
|  | Hoof pigment |  |  | Fleece rot at 75 wks |
|  | LHS fiber |  |  | Fleece rot at 101 wks |
| Lactation | Persistence |  |  | Fleece rot at 154 wks |
|  | Cumulative yield |  |  | Fleece style at 50 wks |
|  | Maximum yield |  |  | Fleece style at 75 wks |
|  | Time to reach maximum yield |  |  | Fleece style at 101 wks |
|  | Time to reach 90% of total yield |  |  | Fleece dag at 75 wks |
|  | Time to reach 75% of total yield |  | Carcass | Final weight |
| Milk Composition | Protein |  |  | Carcass weight |
|  | Fat |  |  | Full CT scan image |
|  | Lactose |  |  | Eye-muscle area |
|  | Somatic cell count |  |  | Lean |
| Wool Analysis | Yield |  |  | Fat |
|  | Micron |  |  | Bone |
|  | FD |  | Body Weight | Week 00 |
|  | SD |  |  | Week 02 |
|  | CV |  |  | Week 10 |
|  | %>30 |  |  | Week 15 |
|  | Comfort |  |  | Week 19 |
|  | SpinF |  |  | Week 24 |
|  | Curv |  |  | Week 25 |
|  | Wool growth/ cm**2 |  |  | Week 32 |
|  | Fibre length |  |  | Week 37 |
|  | Crimp |  |  | Week 43 |
|  | Follicle density |  |  | Week 48 |
|  | Follicle ratio |  |  | Week 50 |
| Feed intake | Feed intake daily |  |  | Week 56 |
|  | Feeding frequency |  |  | Week 60 |
|  | Feeding duration |  |  | Week 67 |
|  | Body weight at feeding |  |  | Week 74 |
|  | Cumulative feed intake |  |  | Week 79 |
|  | Energy partitioning |  |  | Week 83 |
| Behaviour | Flight speed |  |  | Week 90 |
|  | Temperament milking |  |  | Week 98 |
|  | Temperament handling |  |  |  |
