# Supplementary material for: Mapping quantitative trait loci (QTL) in sheep. I. A new male framework linkage map and QTL for growth rate and body weight
Source: Genet Sel Evol. 2009 Apr 24;41(1):34. doi: 10.1186/1297-9686-41-34 (PMC2686678; doi:10.1186/1297-9686-41-34)
Supplement: Additional file 2 — Methodology QTL-MLE. A detailed description of the QTL-MLE methodology which was developed by PCT and was used for the analysis shown here. [file 1297-9686-41-34-S2.doc]

### Additional file 2 - Methodology QTL-MLE

**Model for body weights**

The piecewise-linear model for sheep weights was specified as follows:

$$y_{it}=b_{0}+\beta_{m}\text{Sex}_{i}+\sum_{j=1}^{4} b_{ij}x_{jt}+\sum_{j=8}^{8} b_{ij}\text{Sex}_{i}x_{jt}+\varepsilon_{it}$$

where

*y_it_* = weight of animal *i* at age *t* (kg);

Sex*_i_* = if 1 if animal *i* is male, 0 if female;

*x_j_*_1_ = Age of animal (weeks);

$$x_{jt}=\left\{\begin{matrix} \text{Age}-c_{j} & \text{if Age}-c_{j}\geq0 \\ 0 & \text{otherwise.} \end{matrix}\right.$$

The breakpoints (knots) were set at *c*_1_ = 0, *c*_2_ = 43, *c*_3_ = 56, and *c*_4_ = 83(weeks #0). The regression coefficients *b_i_*_0_, *b_i_*_1_, …, *b_i_*_8_ were modeled as random effects, *b_ij_* = β*_j_* + *B_ij_*; *j* = 0, 1, …, 8, and where the β*_j_* were the overall fixed effects. The random effect deviations were modeled as a multivariate normal distribution,$\mathbf{B}_{i}\sim N(\mathbf{0},\mathbf{\Sigma}_{B})$. To make allowance for any possible serial correlation resulting from the repeated measures data, an exponential correlation structure was fitted to the random errors from within each animal, i.e.,

$$\mathrm{cor}(\varepsilon_{it_{1}},\varepsilon_{it_{2}})=\exp(-\theta|t_{1}-t_{2}|)$$

This is an extension of an autoregressive AR(1) error structure, which takes into account unequally spaced recording times.

All data were included in this analysis, including those only weighed once. The Model fitting was conducted using ASReml.

QTL

Animal M1 M2 M3 M4 M5 M6 M7

1 1 **2 12 12 1** 2 12

2 **12 12 12 1** 12 1 1

3 12 **1 1** 1 12 1 2

…

*d*

Figure: Illustration of the range of different sets of markers that can provide linkage information for a QTL, for three hypothetical backcross animals each genotyped for the paternal allele at seven marker loci M1, . . . , M7. The putative QTL is drawn as the vertical line at position *d*. Genotypes, recorded as ‘1’, ‘2’ or ‘12’, are shown for each marker locus. For each animal, the range of markers that provide information is shown as the horizontal line

**Calculation of QTL transmission probabilities**

To calculate the transmission probabilities, we determine all the possible “pathways” between unambiguous markers (starting at maker *j* = 0 ending at marker *j* = *k* + 1). (So for example for the sequence ‘2–12–12–1’, consider all the possible F_1_ sire and Merino dam gametes that could have produced this.) It can be shown that the resultant transmission probability is

$$\pi=p(q=1|\mathbf{m})=\frac{p(\mathbf{m},q=1)}{p(\mathbf{m})}=\frac{\sum_{i\in\{i: t_{i,k+1}=g_{k+1}\}} \left[\prod_{j=0}^{k} r'_{j,j+1}^{s_{ij}}n'_{j,j+1}^{1-s_{ij}}\prod_{j=1}^{k} p_{j1}^{1-t_{ij}}p_{j2}^{t_{ij}}\right]}{\sum_{i\in\{i: t_{i,k+1}=g_{k+1}\}} \left[\prod_{j=0}^{k} r_{j,j+1}^{s_{ij}}n_{j,j+1}^{1-s_{ij}}\prod_{j=1}^{k} p_{j1}^{1-t_{ij}}p_{j2}^{t_{ij}}\right]}$$

where $r_{j,j+1}$ is the probability of a recombination between markers *j* and *j* + 1 ($n_{j,j+1}=1-r_{j,j+1}$) in the sire gamete; $r'_{j,j+1}=r_{j,j+1}$ if the putative QTL is not flanked by markers *j* and *j* + 1, otherwise it is the probability of recombination between the markers *and* of transmitting QTL allele *Q* (i.e. *q* = 1). To cater for the different pathways, the following indicator variables have been introduced,

$g_{j}=\left\{\begin{matrix} 1 & \text{if marker }j\text{ has genotype '1'} \\ 0 & \text{otherwise (genotype '2')} \end{matrix}\right.$ *j* = 0, *k* + 1;

$$s_{ij}=\left\{\begin{matrix} 1 & \text{if recombination between marker }j\text{ and }j+1\text{ for pathway }i \\ 0 & \text{otherwise;} \end{matrix}\right.$$

and

$$t_{ij}=\left\{\begin{matrix} 1 & \text{if ram transmits allele '1' at locus }j\text{, for pathway }i \\ 0 & \text{otherwise,} \end{matrix}\right.$$

which may be calculated recursively as $t_{ij}=t_{i,j-1}(1-s_{i,j-1})+(1-t_{i,j-1})s_{i,j-1}$, setting *t_i_*_0_ = *g*_0_ as required, depending on the genotype of the first informative marker of the genotype sequence. The terms *p_j_*_1_ and *p_j_*_2_ are the frequencies of alleles ‘1’ and ‘2’ at locus *j* in the Merino population, and maximum likelihood estimates for these can be obtained as follows. Dropping the subscript *j*, let the allele frequency of 1, 2, and all other alleles be *p*_1_, *p*_2_, and *p*_3_, with *p*_3_ = 1 – *p*_1_ – *p*_2_. Also, assume that the observed marker frequencies are *f*_1_, *f*_2_, and *f*_3_ for genotypes 1*x*, 2*x*, and 12 respectively. Then the likelihood (ignoring a constant) for these data is $L=(1-p_{2})^{f_{1}}(1-p_{1})^{f_{2}}(1-p_{3})^{f_{3}}$, for which the maximum likelihood estimates are

$$\begin{matrix} \hat{p}_{1}=0 & \hat{p}_{2}=\frac{f_{3}}{f_{1}+f_{3}} & \hat{p}_{3}=\frac{f_{1}}{f_{1}+f_{3}} & \text{if }f_{1}+f_{3}<f_{2} \\ \hat{p}_{1}=\frac{f_{3}}{f_{2}+f_{3}} & \hat{p}_{2}=0 & \hat{p}_{3}=\frac{f_{2}}{f_{2}+f_{3}} & \text{if }f_{2}+f_{3}<f_{1} \\ \hat{p}_{1}=\frac{f_{1}}{f_{1}+f_{2}} & \hat{p}_{2}=\frac{f_{2}}{f_{1}+f_{2}} & \hat{p}_{3}=0 & \text{if }f_{1}+f_{2}<f_{3} \\ \hat{p}_{1}=\frac{f_{1}-f_{2}+f_{3}}{f_{1}+f_{2}+f_{3}} & \hat{p}_{2}=\frac{f_{2}-f_{1}+f_{3}}{f_{1}+f_{2}+f_{3}} & \hat{p}_{3}=\frac{f_{1}+f_{2}+f_{3}}{f_{1}+f_{2}+f_{3}} & \text{otherwise.} \end{matrix}$$
