# Supplementary material for: Mapping quantitative trait loci (QTL) in sheep. I. A new male framework linkage map and QTL for growth rate and body weight
Source: Genet Sel Evol. 2009 Apr 24;41(1):34. doi: 10.1186/1297-9686-41-34 (PMC2686678; doi:10.1186/1297-9686-41-34)
Supplement: Additional file 3 — LOD score difference between the best and second best map order. This tables shows the differences of the LOD scored between the best and the second best map order, shown are the results on each autosome. [file 1297-9686-41-34-S3.doc]

### Additional file 3- LOD score difference between the best and second best map order

| OAR | LOD Difference |  | OAR | LOD Difference |
| --- | --- | --- | --- | --- |
| 1 | 2.81 |  | 14 | 12.54 |
| 2 | 2.11 |  | 15 | 6.03 |
| 3 | 5.41 |  | 16 | 4.51 |
| 4 | 16.1 |  | 17 | 1.96 |
| 5 | 3.62 |  | 18 | 3.44 |
| 6 | 13.01 |  | 19 | 4.21 |
| 7 | 3.85 |  | 20 | 6.97 |
| 8 | 8.01 |  | 21 | 9.66 |
| 9 | 9.67 |  | 22 | 6.96 |
| 10 | 2.65 |  | 23 | 9.39 |
| 11 | 7.62 |  | 24 | 7.20 |
| 12 | 8.27 |  | 25 | 6.08 |
| 13 | 8.61 |  | 26 | 9.23 |
