# Supplementary material for: Mapping quantitative trait loci (QTL) in sheep. I. A new male framework linkage map and QTL for growth rate and body weight
Source: Genet Sel Evol. 2009 Apr 24;41(1):34. doi: 10.1186/1297-9686-41-34 (PMC2686678; doi:10.1186/1297-9686-41-34)
Supplement: Additional file 4 — Marker information for the Awassi × Merino map. The figures shown here summarize the markers used in the study and compare the framework map described in the Awassi × Merino resource population with the published map. Further the identified linkage regions from the present study and QTL and candidate genes from various references are shown. All figures were designed using the MapChart software described by Voorrips R.E. in 2002 (MapChart: Software for the graphical presentation of linkage maps and QTLs. The Journal of Heredity 93 (1): 77–78). [file 1297-9686-41-34-S4.doc]

### Additional file 4 - Marker information for the Awassi × Merino map

|  |  |
| --- | --- |

|  |  |
| --- | --- |
|  |  |

|  |  |
| --- | --- |

|  |  |
| --- | --- |
|  |  |

|  |  |
| --- | --- |

|  |  |
| --- | --- |
