# Supplementary material for: Mapping quantitative trait loci (QTL) in sheep. I. A new male framework linkage map and QTL for growth rate and body weight
Source: Genet Sel Evol. 2009 Apr 24;41(1):34. doi: 10.1186/1297-9686-41-34 (PMC2686678; doi:10.1186/1297-9686-41-34)
Supplement: Additional file 5 — Summary of results using QTL-MLE and QTL Express. Values in table are estimated QTL positions (cM). This table shows the average QTL positions of all QTL presented here using both programs QTL-MLE and QTL Express. It summarizes and compares all results observed in the resource population for the growth and weight traits. [file 1297-9686-41-34-S5.doc]

### Additional file 5 - Summary of results using QTL-MLE and QTL Express

### Values in table are estimated QTL positions (cM)

| Summary Results QTL-MLE | | | | | | | | | | | | | | | | | | | | | | | | | | |
| --- | --- | --- | --- | --- | --- | --- | --- | --- | --- | --- | --- | --- | --- | --- | --- | --- | --- | --- | --- | --- | --- | --- | --- | --- | --- | --- |
|  | OAR | | | | | | | | | | | | | | | | | | | | | | | | | |
|  | 1 | 2 | 3 | 4 | 5 | 6 | 7 | 8 | 9 | 10 | 11 | 12 | 13 | 14 | 15 | 16 | 17 | 18 | 19 | 20 | 21 | 22 | 23 | 24 | 25 | 26 |
| BW43 | 117 | - | 29 | - | - | 63 | - | - | - | - | 55 | - | - | - | - | - | - | - | - | - | 29 | - | - | 85 | - | - |
| BW56 | - | - | 42 | - | - | 62 | - | - | - | - | 55 | - | - | - | - | - | - | - | - | - | 27 | - | - | 85 | - | - |
| BW83 | 357 | - | 42 | - | - | 60 | - | - | - | - | 55 | - | - | - | - | - | - | - | - | - | 26 | - | - | 86 | - | - |
| BW98 | - | - | - | - | - | - | - | - | - | - | 29 | - | - | - | - | - | - | - | - | - | - | - | 71 | - | - | - |
| GR00-43 | 118 | - | 28 | - | - | 62 | - | 77 | - | - | 55 | - | - | - | - | - | - | - | - | - | 26 | - | - | 86 | - | - |
| GR43-56 | 357 | - | 39 | - | - | 60 | - | - | - | - | 54 | - | - | - | - | - | - | - | - | - | 27 | - | - | 85 | - | 63 |
| GR56-83 | 357 | - | 105 | - | - | 50 | - | - | - | - | 53 | - | - | - | - | - | - | - | - | - | 29 | - | - | 85 | - | 63 |
| GR83-98 | - | - | - | - | - | - | - | - | - | - | 29 | - | - | - | - | - | - | - | - | - | - | - | 40 | - | - | - |
| GR00-43 adj for BW02 | 357 | - | - | - | - | 43 | - | - | - | - | 56 | 104 | - | - | - | 98 | - | - | - | - | 19 | - | - | 91 | - | - |
| GR56-83 adj for BW56 | - | - | 105 | - | - | - | 100 | - | - | - | - | - | - | - | - | - | - | 72 | - | - | - | - | - | - | - | - |
| GR83-98 adj for BW83 | 48 | - | - | - | - | - | - | - | - | - | - | - | - | - | - | - | - | - | - | - | - | - | - | - | - | - |
|  |  |  |  |  |  |  |  |  |  |  |  |  |  |  |  |  |  |  |  |  |  |  |  |  |  |  |
| Summary Results QTL Express | | | | | | | | | | | | | | | | | | | | | | | | | | |
|  | OAR | | | | | | | | | | | | | | | | | | | | | | | | | |
| Traits | 1 | 2 | 3 | 4 | 5 | 6 | 7 | 8 | 9 | 10 | 11 | 12 | 13 | 14 | 15 | 16 | 17 | 18 | 19 | 20 | 21 | 22 | 23 | 24 | 25 | 26 |
| BW43 | - | - | - | - | - | 56 | - | - | - | - | 48 | - | - | - | - | - | - | - | - | - | 68 | - | - | 68 | - | - |
| BW56 | - | - | - | - | - | 56 | - | - | - | - | 48 | - | - | - | - | - | - | - | - | - | 52 | - | - | 68 | - | - |
| BW83 | - | - | - | - | - | 56 | - | - | - | - | 28 | - | - | - | - | - | - | - | - | - | 28 | - | - | 72 | - | - |
| BW98 | 340 | - | 56 | - | - | - | - | - | - | - | 0 | - | - | - | - | - | - | - | - | - | - | 80 | 68 | - | - | - |
| GR00-43 | - | - | - | - | - | 44 | - | - | - | - | 52 | - | - | - | - | - | - | - | - | - | 68 | - | - | - | - | - |
| GR43-56 | - | - | - | - | - | 56 | - | - | - | - | 28 | - | - | - | - | - | - | - | - | - | 28 | - | - | 72 | - | - |
| GR56-83 | - | - | 296 | - | - | 56 | - | - | - | - | 24 | - | - | - | - | - | - | - | - | - | 28 | - | - | - | - | 60 |
| GR83-98 | 344 | - | 292 | - | - | - | - | - | - | - | 0 | - | - | - | 20 | 56 | - | - | - | - |  | 84 | 68 | - | - | - |
| GR00-43 adj for BW02 | - | - | - | - | - | 36 | - | - | - | - | 52 | - | - | - | - | 96 | - | - | - | - | - | - | - | - | - |  |
| GR43-56 adj for BW43 | - | - | 104 | - | - | - | - | - | - | - | - | - | - | - | - | - | - | - | - | - | - | - | - | - | - | 56 |
| GR83-98 adj for BW83 | 24 | - | - | - | - | - | - | - | - | - | - | - | - | - | - | - | - | - | - | - | - | - | - | - | - | - |
| GR00-43 adj for BW02* | - | - | - | 108 112 | - | - | - | - | - | - | - | - | - | - | - | - | - | - | - | - | - | - | - | - | - | - |
| GR56-83 adj for BW56* | - | - | 104 284 | - | - | - | - | - | - | - | - | - | - | - | - | - | - | - | - | - | - | - | - | - | - | - |
| GR83-98 adj for BW83* | - | - | - | - | - | - | - | - | - | - | - | - | - | - | - | - | - | - | - | - | - | 68 88 | - | - | - | - |

* Two-QTL models
