# Supplementary material for: Mapping quantitative trait loci (QTL) in sheep. I. A new male framework linkage map and QTL for growth rate and body weight
Source: Genet Sel Evol. 2009 Apr 24;41(1):34. doi: 10.1186/1297-9686-41-34 (PMC2686678; doi:10.1186/1297-9686-41-34)
Supplement: Additional file 6 — 3D surface plot of the variance ratio test statistic for a two-QTL model. This figure shows the 3D surface plot for a two-QTL model for GR56-83 adj for BW56 on OAR3. The values of the plot are generated by QTL Express. [file 1297-9686-41-34-S6.doc]

### Additional file 6 – 3D surface plot of the variance ratio test statistic for a two-QTL model for GR56-83 adj for BW56 on OAR3
