# Supplementary material for: Mapping quantitative trait loci (QTL) in sheep. I. A new male framework linkage map and QTL for growth rate and body weight
Source: Genet Sel Evol. 2009 Apr 24;41(1):34. doi: 10.1186/1297-9686-41-34 (PMC2686678; doi:10.1186/1297-9686-41-34)
Supplement: Additional file 7 — Comparison of the QTL analysis results in the present studies with other results and possible candidate genes. This table describes QTL identified in other studies which are located in comparable regions to the linkage regions identified using animals of the Awassi × Merino population. Further possible candidate genes are described. The list of references for these data is attached following the table. [file 1297-9686-41-34-S7.doc]

### Additional file 7 - Comparison of the QTL analysis results in the present studies with other results and possible candidate genes

| OAR | QTL presented here | Position (marker) | Other QTL published | Possible candidate gene | Literature |
| --- | --- | --- | --- | --- | --- |
| OAR1 | GR00-43adj, GR56-83, GR43-56, BW83 | 357 cM (BMS1789) |  | transferrin (TF) gene | [1, 2] |
|  | GR00-43adj | OAR1q21-22 309 cM (BM7145, BM864) | QTL for weight and slaughter live weight | POU domain, class 1 transcription factor 1 (POU1F1), | [3-6] |
| OAR2 |  | q-arm | QTL for slaughter live weight | Myostatin | [2, 5] |
| OAR3 |  | (OARVH130, BMS1248) | growth QTL (cattle) | insulin-like growth factor 1 (IGF1) | [2, 7, 8] |
| OAR6 | GR00-43adj | (MCM204) | birth weight variability (humans) | the epidermal growth factor (EGF) gene | [9, 10] |
| OAR11 | Different, e.g. BW43 | (EPCDV23) | Associations to growth (cattle); greater abundance of GH mRNA in the pituitary somatotropes from genetically lean sheep | growth hormone 1 (GH1) | [2, 11, 12] |
| OAR18 | adjusted growth rate week 56-83 |  |  | callipyge locus, rib-eye muscling locus, Carwell locus | [2, 13, 14] |
| OAR23 | growth rate week 83, weight week 98 |  | associated with energy expenditure and weight gain | Melanocortin 4 receptor (MC4R) | [15, 16] |
| OAR24 | BW43 to 83, GR00-83 | 79 cM | FBS1 expressed in muscle, it induces fibroblast proliferation | erythropoietin(EPO #0), elsatin (ELN), fibrosin (FBS1) | [17] |
| OAR26 | growth rate | 50 cM (CSSM043, OARJMP23) | associated with obesity and metabolic disease (human) | beta-3-adrenergic receptor (ADRB3) | [18] |

1. Kmiec M: **Transferrin Polymorphism versus Growth Rate in Lambs, Polish Long-wool Sheep - II. Analysis of relation between transferrin polymorphism of lamb blood serum versus growth rate of lambs up to age of 5 months**. *Archiv Fur Tierzucht-Archives of Animal Breeding* 1999, **42**(5):469-479.

2. Walling GA, Visscher PM, Wilson AD, McTeir BL, Simm G, Bishop SC: **Mapping of quantitative trait loci for growth and carcass traits in commercial sheep populations**. *Journal of Animal Science* 2004, **82**(8):2234-2245.

3. Woollard J, Tuggle CK, de Leon FAP: **Rapid communication: Localization of POU1F1 to bovine, ovine, and caprine 1q21-22**. *Journal of Animal Science* 2000, **78**(1):242-243.

4. Jiang R, Li J, Qu L, Li H, Yang N: **A new single nucleotide polymorphism in the chicken pituitary-specific transcription factor (POU1F1) gene associated with growth rate**. *Animal Genetics* 2004, **35**(4):344-346.

5. Karamichou E, Richardson RI, Nute GR, McLean KA, Bishop SC: **A partial genome scan to map quantitative trait loci for carcass composition, as assessed by X-ray computer tomography, and meat quality traits in Scottish Blackface Sheep**. *Animal Science* 2006, **82**:301-309.

6. McRae AF, Bishop SC, Walling GA, Wilson AD, Visscher PM: **Mapping of multiple quantitative trait loci for growth and carcass traits in a complex commercial sheep pedigree**. *Animal Science* 2005, **80**:135-141.

7. Andrade PC, Grossi DA, Paz CCP, Alencar MM, Regitano LCA, Munari DP: **Association of an insulin-like growth factor 1 gene microsatellite with phenotypic variation and estimated breeding values of growth traits in Canchim cattle**. *Animal Genetics* 2008.

8. Stone RT, Keele JW, Shackelford SD, Kappes SM, Koohmaraie M: **A primary screen of the bovine genome for quantitative trait loci affecting carcass and growth traits**. *J Anim Sci* 1999, **77**(6):1379-1384.

9. Dissanayake VHW, Tower C, Broderick A, Stocker LJ, Seneviratne HR, Jayasekara RW, Kalsheker N, Pipkin FB, Morgan L: **Polymorphism in the epidermal growth factor gene is associated with birthweight in Sinhalese and white Western Europeans**. *Molecular Human Reproduction* 2007, **13**(5-6):425-429.

10. Lord EA, Lumsden JM, Dodds KG, Henry HM, Crawford AM, Ansari HA, Pearce PD, Maher DW, Stone RT, Kappes SM *et al*: **The linkage map of sheep chromosome 6 compared with orthologous regions in other species**. *Mammalian Genome* 1996, **7**(5):373-376.

11. Phillips JA: **Inherited defects in growth hormone synthesis and action**. In: *The Metabolic and Molecular Bases of Inherited Disease.* vol. II. New York: McGraw-Hill; 1995: 3023-3044.

12. Taylor JF, Coutinho LL, Herring KL, Gallagher DS, Brenneman RA, Burney N, Sanders JO, Turner RV, Smith SB, Miller RK *et al*: **Candidate gene analysis of GH1 for effects on growth and carcass composition of cattle**. *Animal Genetics* 1998, **29**(3):194-201.

13. Cockett NE, Jackson SP, Shay TL, Nielsen D, Moore SS, Steele MR, Barendse W, Green RD, Georges M: **Chromosomal localization of the callipyge gene in sheep (Ovis aries) using bovine DNA markers**. *Proceedings of the National Academy of Sciences of the United States of America* 1994, **91**(8):3019-3023.

14. Nicoll GB, Burkin HR, Broad TE, Jopson NB, Greer GJ, Bain WE, Wright CS, Dodds KG, Fennessy PF, McEwan JC: **Genetic linkage of microsatellite markers to the Carwell locus for rib-eye muscling in sheep**. In: *Proc 6th World Cong Genet Appl Livest: 1998*; 1998.

15. Jaakola L, Pirttila AM, Vuosku J, Hohtola A: **Method based on electrophoresis and gel extraction for obtaining genomic DNA-free cDNA without DNase treatment**. *Biotechniques* 2004, **37**(5):744-+.

16. Rutanen J, Pihlajamaki J, Karhapaa P, Vauhkonen I, Kuusisto J, Moilanen Mykkanen L, Laakso M: **The Val103Ile Polymorphism of Melanocortin-4 Receptor Regulates Energy Expenditure and Weight Gain[ast][ast]**. *Obesity* 2004, **12**(7):1060-1066.

17. Prakash S, Robbins PW: **Cloning and analysis of the cDNA for human fibrosin, a novel fibrogenic lymphokine**. *DNA and Cell Biology* 1998, **17**(10):879-884.

18. Connor EE, Ashwell MS, Schnabel R, Williams JL: **Comparative mapping of bovine chromosome 27 with human chromosome 8 near a dairy form QTL in cattle**. *Cytogenetic and Genome Research* 2006, **112**(1-2):98-102.
